# Worsening Endothelial Function with Efavirenz Compared to Protease Inhibitors: A 12-Month Prospective Study

**DOI:** 10.1371/journal.pone.0045716

**Published:** 2012-09-20

**Authors:** Samir K. Gupta, Changyu Shen, Sharon M. Moe, Lisa M. Kamendulis, Mitchell Goldman, Michael P. Dubé

**Affiliations:** 1 Division of Infectious Diseases, Department of Medicine, Indiana University School of Medicine, Indianapolis, Indiana, United States of America; 2 Department of Biostatistics, Indiana University School of Medicine, Indianapolis, Indiana, United States of America; 3 Division of Nephrology, Department of Medicine, Indiana University School of Medicine, Indianapolis, Indiana, United States of America; 4 Department of Environmental Health, Indiana University, Bloomington, Indiana, United States of America; 5 Division of Infectious Diseases, Department of Medicine, University of Southern California Keck School of Medicine, Los Angeles, California, United States of America; University of Washington, United States of America

## Abstract

**Objective:**

Changes in endothelial function, measured as flow-mediated dilation (FMD) of the brachial artery, has not been systematically assessed beyond 6 months of initiation of antiretroviral therapy (ART) when drug-related effects might offset initial improvements with virologic control.

**Design:**

We assessed 6 and 12 month changes in FMD [presented as median (quartile 1, quartile 3)] and circulating HIV and cardiovascular biomarkers in 23 subjects initiating ART.

**Results:**

There were no significant changes in FMD at 6 or 12 months overall despite significant increases in CD4 cell count and HDL-C and reductions in HIV RNA level, MCP-1, IP-10, sVCAM-1, sTNFR2, and sCD14. However, there were significant differences (P = 0.04) in the changes in FMD between those receiving efavirenz [N = 12; −3.50% (−4.90%, 0.68%)] vs. protease inhibitors at 12 months [N = 11; 1.50% (−0.86%, 4.56%)]. The differences in changes in FMD between those receiving and not receiving emtricitabine/tenofovir/efavirenz were more pronounced and were significantly different at both 6 and 12 months (P<0.02 for both). Additional studies showed no significant differences in changes in 25-(OH)-vitamin D, PTH, FGF-23, of F2-isoprostane levels between efavirenz and PI use or between those receiving and not receiving emtricitabine/tenofovir/efavirenz.

**Conclusion:**

Efavirenz use was associated with reduced FMD at 12 months compared to PI-based regimens while emtricitabine/tenofovir/efavirenz was associated with reduced FMD at both 6 and 12 months compared to those not receiving this combination. Long-term effects of antiretrovirals on endothelial function may play an important role in the risk of cardiovascular disease in HIV-infected patients.

## Introduction

It has been suggested that both HIV infection itself and the antiretroviral therapies (ART) used to treat HIV contribute to the increased risk of cardiovascular disease (CVD) seen in this population [1]. Impaired endothelial function is a key initial step in the development of atherosclerosis [2]. In ACTG 5152s, endothelial function, as measured by flow-mediated dilation (FMD) of the brachial artery, improved with antiretroviral therapy, regardless of its component drugs or associated lipid abnormalities, over the first six months of treatment [3]. Thus, controlling HIV replication appears to be a key factor in the initial increases in FMD seen with ART initiation. However, other studies suggest that use of specific antiretrovirals, such as abacavir, may contribute to lower FMD [4] and with eventual CVD [5]. However, in ACTG 5202 there were a surprisingly greater number of ischemic events over the first two years of the trial in those receiving tenofovir/emtricitabine/efavirenz compared to those in the other treatment groups [6]. Thus, it seems plausible to hypothesize that any initial improvements in FMD with viral load reduction may subsequently be negated by ART-related toxic effects. In addition, is seems likely that specific ART regimens may lead to greater CVD risk than others. We examined these possibilities in a prospective, 12-month study assessing changes in FMD in 23 subjects initiating ART.

## Methods

### Study Design

A total of 23 antiretroviral-naïve, HIV-infected subjects were enrolled into this pilot study to assess the effects of ART on endothelial function over 12 months and to assess possible predictors and correlates of changes in FMD over this time period. These subjects were recruited from the HIV treatment clinics at the Indiana University School of Medicine if they appeared to meet the eligibility criteria for study participation. Because no specific patient groups were otherwise targeted for study inclusion, the study cohort was comparable to the characteristics of our general clinic population initiating ART. Although the choice of antiretrovirals was left to the subjects’ providers, we a priori specified a comparison of changes in FMD between those receiving and not receiving protease inhibitors. FMD and nitroglycerin-mediated dilation (NTGMD) were measured according to established methods as we have previously published [7] at baseline prior to ART initiation, after six months, and after twelve months of treatment. All ultrasound procedures were performed by a single registered vascular technician. All vascular measurements were made by a single investigator (S.K.G.) using AccessPoint 2004 software (Freeland Systems, Inc., Indianapolis, IN) who was not aware of subject characteristics or treatments. The intraclass correlations for reproducibility for baseline diameter and FMD measured twice in 12 healthy volunteers in our laboratory under these conditions were 0.97 and 0.73, respectively, which are similar to those reported from other productive vascular imaging laboratories [8].

HIV parameters (CD4 cell count, HIV-1 RNA level) were measured at each visit. Markers of inflammation [monocyte chemoattractant protein-1 (MCP-1), interferon-inducible protein-10 (IP-10), high sensitivity C-reactive protein (hsCRP), interleukin-6 (IL-6), soluble tumor necrosis factor receptor 2 (sTNFR2), soluble vascular cell adhesion molecule-1 (sVCAM-1) and bacterial translocation [soluble CD14 (sCD14)] were measured using a multiplex assay from Aushon Biosystems Inc. (Woburn, MA). Metabolic parameters (lipid fractions, glucose, insulin) were measured using standard methods at the Indiana University Health and Clinical Research Center laboratories. Markers of mineral metabolism were measured by ELISA [25-OH-vitamin D (ALPCO, Salem, NH)], parathyroid hormone (PTH; ALPCO, Salem, NH), fibroblast growth factor-23 (FGF-23; Immuntopics, San Clemente, CA)]. F2-isoprostanes, a marker of oxidative stress, were measured using a standard LC-MSMS assay [9]. All samples were measured in batch from archived specimens obtained at these study visits; these samples were frozen at −80°C and thawed just once for these measurements. All assessments were made after an eight-hour fast.

### Eligibility Criteria

All subjects were at least 18 years of age, had documented HIV infection, and had been free of antiretroviral treatment for at least six months prior to screening. Subjects were excluded for known vascular disease, diabetes, hypertension, pro-inflammatory conditions (other than HIV or hepatitis B or C co-infection), history of malignancy, fever or recent treatment of infection, or current receipt of other anti-inflammatory, investigational, or lipid-lowering drugs.

### Statistical Analysis

Continuous variables are presented as medians (quartile 1, quartile 3) unless otherwise specified. Categorical variables are summarized by frequency and percentage. Changes in FMD, NTGMD, and laboratories were assessed using the Signed-Rank test with comparisons between groups assessed using the Wilcoxon Rank-Sum test. Because FMD is heavily dependent on baseline brachial artery diameter and heart rate, changes in FMD were adjusted for these two variables using a linear mixed-effects model with random intercepts. All FMD and NTGMD results were complete for all 23 subjects, but there were 1 or 2 missing data points missing at various time points for each of the biomarker data except for the F2-isoprostane results for which there were 3 missing data points at baseline and for the FGF-23 results for which there were 4 missing data point at baseline. All of the biomarker analyses were performed only for those subjects for whom complete longitudinal data were available, i.e. no imputation methods were applied. In addition, correction for multiple testing was not performed in this preliminary study. All tests were 2-sided with p-values less than 0.05 considered significant. All analyses were performed in SAS 9.2 (SAS Inc., Cary, NC).

### Ethics Statement

This study was approved by the Indiana University School of Medicine Institutional Review Board. All participants provided written, informed consent.

## Results

Subject characteristics and the levels of FMD, NTGMD, and circulating biomarkers throughout the study period are shown in Table 1. Efavirenz-based ART was used throughout the study period in 12 subjects and the remainder used protease inhibitor (PI)-based regimens (6 received atazanavir/ritonavir and 4 received lopinavir/ritonavir throughout the study; 1 other subject initially received atazanavir/ritonavir through the 6 month visit and then switched to lopinavir/ritonavir). Thirteen subjects received tenofovir/emtricitabine, 5 subjects received abacavir/lamivudine, and 5 others received other nucleoside combinations. Specifically, 8 subjects received tenofovir/emtricitabine/efavirenz.

**Table 1:** Baseline Characteristics and Measures of Endothelial Function, Metabolic Parameters, Inflammation, Bacterial Translocation, and Oxidative Stress in the Overall Study Cohort (N = 23) and in the Efavirenz (N = 12) and Protease Inhibitor (N = 11) Subgroups.

Characteristic	Sub-characteristic	Baseline	Six Months	Twelve Months
Age, years	Overall	39.6 (33.5, 46.4)		
	EFV	43.2 (39.3, 46.7)		
	PI	35.1 (25.6, 39.7)		
Male sex, n (%)	Overall	21 (91)		
	EFV	10 (83)		
	PI	11 (100)		
Black race, n (%)	Overall	8 (35)		
	EFV	4 (33)		
	PI	4 (36)		
CD4 cell count/µL	Overall	216 (73, 296)	258 (191, 572); p<0.0001	342 (190, 589); p<0.0001
	EFV	235 (151,278)	292 (207, 474); p = 0.002	385 (275, 525); p = 0.001
	PI	159 (48, 390)	242 (190, 727); p = 0.002	228 (150, 642); p = 0.04
HIV-1 RNA log_10_copies/mL	Overall	5.00 (4.39, 5.31)	1.78 (1.70, 2.60); p<0.0001	1.70 (1.68, 2.08); p<0.0001
	EFV	4.97 (4.30, 5.08)	1.70 (1.70, 2.60); p = 0.0005	1.68 (1.68, 1.85); p = 0.0005
	PI	5.24 (4.71, 5.37)	1.98 (1.70, 3.78); p = 0.002	1.78 (1.70, 2.72); p = 0.001
FMD, %	Overall	5.86 (2.16, 7.35)	4.67 (2.86, 6.53)	3.41 (1.71, 7.59)
	EFV	6.81 (1.79, 7.57)	4.32 (2.46, 6.77)	3.12 (1.88, 5.27); p = 0.08
	PI	4.47 (2.85, 7.35)	5.53 (3.62, 6.53)	5.81 (1.55, 7.73)
NTGMD, %	Overall	17.2 (13.5, 22.6)	16.4 (10.3, 21.7)	14.1 (10.2, 22.5)
	EFV	14.4 (12.0, 17.2)	13.0 (10.3, 18.78)	13.2 (10.0, 15.3)
	PI	22.4 (17.2, 25.8)	18.1 (6.1, 21.7)	21.7 (10.2, 24.6)
Total cholesterol, mg/dL	Overall	148 (100,166)	132 (117, 174)	139 (115, 181)
	EFV	162 (110,173)	153 (123, 190)	160 (135, 182)
	PI	160 (135, 182)	126 (104, 132)	131 (105, 146)
LDL-cholesterol, mg/dL	Overall	72 (52, 111)	79 (67, 107)	87 (65, 110)
	EFV	93 (54, 112)	103 (75, 114)	96 (66, 118)
	PI	63 (47, 111)	74 (50, 79)	78 (58, 90)
HDL-cholesterol, mg/dL	Overall	37 (27, 46)	47 (43, 57); p<0.0001	46 (38, 62); p<0.0001
	EFV	43 (29, 48)	56 (46, 63); p = 0.002	56 (39, 71); p = 0.002
	PI	32 (23, 42)	44 (41, 48); p = 0.01	43 (35, 55); p = 0.005
Triglycerides, mg/dL	Overall	116 (88, 178)	132 (91, 169)	125 (84, 177)
	EFV	154 (87, 185)	106 (90, 147)	100 (85, 168)
	PI	100 (88, 178)	164 (130, 176)	128 (83, 177)
HOMA-IR	Overall	2.26 (1.84, 3.60)	3.12 (2.28, 4.04); p = 0.03	2.70 (1.93, 4.01); p = 0.06
	EFV	2.75 (2.00, 3.73)	3.26 (2.45, 3.93)	2.92 (2.24, 3.61)
	PI	2.26 (1.41, 2.53)	2.98 (2.21, 4.04)	2.00 (1.79, 4.59); p = 0.07
hsCRP, mg/L	Overall	0.80 (0.30, 1.21)	0.78 (0.19, 1.67)	0.55 (0.29, 1.18)
	EFV	0.79 (0.46, 1.15)	1.07 (0.78, 3.02); p = 0.04	0.58 (0.48, 1.35)
	PI	0.66 (0.16, 3.22)	0.18 (0.12, 0.69); p = 0.07	0.55 (0.12, 0.64)
IL-6, pg/mL	Overall	6.1 (4.8, 7.4)	4.8 (3.3, 7.6); p = 0.10	4.9 (3.6, 7.5)
	EFV	5.7 (4.2, 6.8)	4.7 (3.7, 6.9)	4.5 (3.6, 6.4)
	PI	6.6 (4.8, 9.8)	5.2 (3.0, 8.4)	5.7 (3.9, 8.1)
MCP-1, pg/mL	Overall	771 (605, 1015)	508 (415, 645); p = 0.0002	547 (416, 673); p<0.0001
	EFV	657 (502, 946)	441 (386, 571); p = 0.01	554 (396, 660); p = 0.02
	PI	983 (626, 1338)	560 (498, 785); p = 0.04	547 (475, 846); p = 0.004
IP-10, pg/mL	Overall	379 (270, 495)	119 (94,189); p<0.0001	140 (97, 167); p<0.0001
	EFV	402 (258, 441)	116 (87, 155); p = 0.0005	126 (79, 151); p = 0.0005
	PI	357 (289, 504)	181 (102, 201); p = 0.004	165 (115, 194); p = 0.004
sVCAM-1, µg/mL	Overall	3.3 (2.6, 4.9)	2.4 (1.9, 3.4); p = 0.001	2.1 (1.1, 3.3); p<0.0001
	EFV	3.4 (2.5, 8.5)	2.3 (1.5, 3.2); p = 0.02	1.7 (1.0, 2.7); p = 0.002
	PI	3.3 (2.9, 4.1)	2.8 (2.0, 4.1); p = 0.07	2.7 (1.6, 3.4); p = 0.03
sTNFR2, pg/mL	Overall	3075 (1905, 4042)	1946 (1304, 2263); p<0.0001	1637 (1253, 2012); p<0.0001
	EFV	3032 (1801, 4134)	1771 (1182, 2083); p = 0.001	1560 (1112, 1658); p = 0.0005
	PI	3075 (2387, 3816)	2093 (1626, 2326); p = 0.01	2012 (1337, 2334); p = 0.002
sCD14, µg/mL	Overall	4.7 (2.8, 5.3)	3.9 (2.8, 4.4); p<0.0001	3.6 (2.9, 3.9); p<0.0001
	EFV	4.7 (2.4, 5.8)	3.9 (3.3, 4.9); p = 0.0005	3.6 (2.9, 3.9); p = 0.0005
	PI	4.4 (3.3, 4.9)	3.3 (2.7, 4.1); p = 0.002	3.6 (2.9, 4.3); p = 0.001
25-OH-vitamin D, ng/mL	Overall	18.2 (12.1, 43.6)	19.2 (11.4, 42.1)	22.9 (8.7, 37.7); p = 0.10
	EFV	17.5 (10.9, 52.8)	15.6 (9.4, 61.1)	20.1 (9.0, 39.7)
	PI	20.9 (16.0, 43.6)	20.9 (16.0, 43.6)	29.3 (7.4, 37.7)
PTH, pg/mL	Overall	34.7 (24.2, 42.3)	50.4 (39.3, 59.4); p<0.0001	60.1 (47.5, 70.8); p<0.0001
	EFV	36.5 (29.3, 45.6)	44.5 (38.2, 59.8); p = 0.005	63.7 (48.7, 74.3); p = 0.002
	PI	29.1 (17.3, 38.4)	52.7 (47.7, 56.3); p = 0.008	58.6 (47.5, 63.5); p = 0.002
FGF-23, pg/mL	Overall	7.53 (6.04, 9.63)	7.05 (4.72, 10.80)	7.48 (4.69, 9.95)
	EFV	7.78 (5.33, 11.68)	7.09 (4.88, 11.01)	6.50 (4.11, 11.09)
	PI	7.35 (6.29, 8.05)	6.35 (4.60, 8.93)	7.75 (5.95, 9.51)
F2-isoprostane, pg/mL	Overall	60.4 (42.8, 73.4)	54.6 (39.6, 74.4)	59.7 (41.7, 76.2); p = 0.080
	EFV	54.0 (37.9, 74.1)	50.6 (38.5, 75.4)	56.7 (44.0, 81.3); p = 0.052
	PI	60.9 (52.8, 69.1)	60.0 (46.0, 74.4)	64.7 (46.7, 73.8)

NOTES: Data presented as medians (quartile 1, quartile 3) unless otherwise specified; all p-values are for within-individual changes from baseline (only those ≤0.10 are shown).

ABBREVIATIONS: EFV, efavirenz; PI, protease inhibitor; FMD, flow-mediated dilation; NTGMD; nitroglycerin-mediated dilation; LDL, low-density lipoprotein; HDL, high-density lipoprotein; HOMA-IR, homeostatic model assessment-insulin resistance; hsCRP, high sensitivity C-reactive protein; IL-6, interleukin-6; MCP-1, monocyte chemoattractant protein-1; sTNFR2, soluble tumor necrosis factor receptor-2; IP-10, interferon gamma inducible protein-10; sVCAM-1, soluble vascular cell adhesion molecule-1; sCD14, soluble cluster of differentiation-14; PTH, parathyroid hormone; FGF-23, fibroblast growth factor-23.

As expected, CD4 cell counts and HIV-1 RNA levels improved throughout the study period in the entire cohort. As shown in Table 1, a few of the participants had HIV-1 RNA levels >400 copies/mL at months 6 and 12. The p-values for the Pearson correlation coefficients between HIV-1 RNA level and FMD at baseline, 6 months, and 12 months were 0.7, 0.4, and 0.5, respectively. As such, it is unlikely that the few subjects with detectable viral loads would have confounded our analyses. In the entire cohort, HDL-C also significantly increased from baseline at both 6 and 12 months while the inflammatory markers MCP-1, IP-10, and sTNFR2 all significantly declined at both study time points. The endothelial activation marker sVCAM-1 also significantly declined at 6 and 12 months, and the bacterial translocation marker sCD14 significantly declined only at 12 months in the entire cohort. None of the other measured markers changed over the study period.

Neither FMD nor NTGMD significantly changed over 6 or 12 months in the entire cohort. Of note, there were no significant differences between baseline FMD or NTGMD in those initiated on efavirenz or PI. There were no differences in the changes in NTGMD between these groups at 6 or 12 months. There were no significant differences in the changes in FMD within or between these groups at 6 months. However, at 12 months we observed non-significant worsening of FMD with EFV [−3.50% (−4.90%, 0.68%); P = 0.08] and non-significant improvements in FMD with PI [1.50% (−0.86%, 4.56%); P = 0.3]; the difference in FMD changes at 12 months between these groups was significant (P = 0.04, linear mixed-effects model accounting for baseline diameter and heart rate).

When assessing those receiving tenofovir/emtricitabine/efavirenz specifically, there were non-significant declines at 6 [−2.86% (−5.81%, 0.08%); P = 0.056] and 12 months [−2.19% (−5.13%, 0.75%); P = 0.14]. Those not receiving this combination had non-significant increases in FMD at 6 months [1.85% (−0.37%, 4.07%); P = 0.099] and significant increases at 12 months [2.27% (0.003%, 4.54%); P = 0.0497]. There were significant differences in the changes in FMD between these two groups at both 6 and 12 months (P<0.02 for both).

None of the measured markers of inflammation, metabolism, or bacterial translocation differentially changed between the efavirenz and PI groups or between those receiving and not receiving tenofovir/emtricitabine/efavirenz. Thus, we then considered other potential mechanisms that might result in worsening FMD in those receiving efavirenz and/or tenofovir/emtricitabine. We measured vitamin D and PTH levels [10] as abnormalities in these variables have been associated with efavirenz and tenofovir use [11] and may also adversely affect endothelial function [12], [13] in the general population. We also measured serum FGF-23 levels as this bone-derived hormone is a mediator of renal phosphate wasting and stimulated by vitamin D and PTH [14] and has also been linked to cardiovascular disease [15]. Circulating 25-OH-vitamin D and FGF-23 levels did not significantly change in the entire cohort or in any of the treatment subgroups. However, PTH increased at 6 and 12 months (P<0.0001 for both) in the entire cohort, which was driven primarily by significant increases in those receiving TDF compared to non-TDF regimens (P<0.0001). There were no significant changes in PTH within or between the subgroups receiving efavirenz vs. PI or in those receiving or not receiving tenofovir/emtricitabine/efavirenz.

We then measured circulating F2-isoprostane levels because efavirenz has been previously linked to worsening oxidative stress [16] and because oxidative stress may also lead to endothelial dysfunction [17]. F2-isoprostanes increased with efavirenz at 12 months [3.15 pg/mL (0.20, 6.53); P = 0.052]; however, there were no significant changes in those receiving PI. There were neither significant differences in the changes of F2-isoprostanes between these groups nor in those specifically receiving tenofovir/emtricitabine/efavirenz vs. other combinations during the study period.

## Discussion

These data suggest that efavirenz-containing regimens may lead to more impaired endothelial function over 12 months compared to those containing PI. In fact, differences in FMD at 6 months between these two groups were becoming apparent at 6 months, albeit not significantly so. This is in contrast to the findings of Torriani et al. who found that efavirenz and PI-based regimens had similar improvements in FMD over 6 months [3]. The sample size in our study might not have been sufficient to find significant improvements with all therapies. However, the apparent contradiction in these results does not appear to be due to differences in baseline demographics, CD4 cell counts, or HIV-1 RNA levels between the two studies. Overall, the baseline FMD values in our study vs. those in Torriani’s study may reflect possible differences in performance and reading of the ultrasound measurements, which makes comparisons across FMD studies difficult. It is also possible that perhaps the differences were due to the fact that few of the subjects in ACTG 5152s specifically received tenofovir/emtricitabine/efavirenz. Of course, there may be unknown treatment indication biases in our observational study that would not have occurred in the randomized study by Torriani et al. On the other hand, our results are more closely aligned to a small study [18] of 9 subjects initiating efavirenz-based regimens (7 of which also received tenofovir/emtricitabine) where FMD significantly declined after one month from 8.7% to 4.6% and remained at 5.1% at 6 months post-initiation. Regression to the mean might in our study might also be postulated, especially since FMD was non-statistically greater at baseline in those receiving EFV compared to PI, but the trend lines in median FMD of the two treatment group ‘crossed’ at 6 months and did not meet at the same final value at 12 months.

Large observational studies, including the D:A:D study, have implicated the use of protease inhibitors with greater risk of CVD events [19]. Efavirenz was not found to be associated with CVD events in the D:A:D cohort. However, a large case-control study from Quebec suggested an increased risk of acute myocardial infarction with the use of efavirenz as well with the use of the protease inhibitor lopinavir [20]. These observational cohort data though may include unmeasured confounders that would perhaps nullify these associations. In contrast, the recently published results from ACTG 5202, which randomly assigned subjects to 4 commonly used, once-daily antiretroviral regimens suggested new associations between specific ART and CVD [6]. In this 96-week trial, the overall ischemic event rate was low (total of 11 amongst the 1857 study participants in all four arms). However, there were numerically more acute ischemic events in patients receiving efavirenz (N = 8/929) than in those receiving atazanavir/ritonavir (N = 3/928). In fact, most ischemic events occurred in those specifically receiving emtricitabine/tenofovir/efavirenz (N = 6/464) compared to those not receiving this regimen (N = 5/1393); by our calculation (not reported by the ACTG 5202 investigators), this would appear to be a possibly statistically significant difference (unadjusted P = 0.033 by Fisher’s Exact Test). Thus, our FMD data may possibly provide a mechanistic understanding for the results from ACTG 5202. Moreover, absolute changes in FMD of greater than 3%, like that found in our study, have been strongly associated with future CVD events in several studies in the general population [21], [22].

We did not find that changes in markers of inflammation, lipids, insulin resistance, bacterial translocation, or mineral homeostasis suggested an underlying etiology for why FMD declined preferentially with efavirenz vs. PI. However, we did find steady and nearly significant increases in F2-isoprostane levels over 12 months in those receiving efavirenz; however, there were no between-group differences with those receiving PI. Some [16], although not all [23], cross-sectional studies have suggested that the use of efavirenz can lead to worsening oxidative stress as measured by serum F2-isoprostane levels. Moreover, a recent in vitro study suggested that EFV increases vascular permeability through induction of reactive oxygen species and subsequent reductions in tight junction proteins [24]. Interestingly, the use of atazanavir in diabetic subjects without HIV infection was associated with increased FMD, possibly through the induction of hyperbilirubinemia as an oxidative stress antagonist [25]. In addition, data from our group and others have suggested use of lopinavir/ritonavir may also be associated with improved endothelial function [26], [27], which is substantiated by the current data.

In conclusion, efavirenz-based ART, especially those using a tenofovir/emtricitabine backbone, may worsen endothelial function compared to PI-based regimens at 12 months of therapy despite the clear improvements in inflammatory and endothelial activation markers. However, these results should be interpreted cautiously given that we cannot exclude the possibilities that both Types I and II errors may have occurred due to the numerous statistical tests performed in this small study and to the lack of complete data for the biomarker analyses. Larger studies are thus clearly warranted to further investigate and corroborate these findings and determine the long-term effects of specific antiretrovirals and antiretroviral combinations on vascular function and disease.
